# Systematic Review and Meta‐Analysis: Association Between Circulating and Tissue Levels of Selenium and Zinc and Breast Cancer Risk

**DOI:** 10.1155/tbj/7448703

**Published:** 2026-07-02

**Authors:** Adrienne Pratti Lucarelli, Maria Marta Martins, Mariana Mie Teruya, Thaís Tiemi Yauti, Daniela Rie Hayashi

**Affiliations:** ^1^ Santa Casa de Sao Paulo School of Medical Sciences, São Camilo University Center, São Paulo, Brazil, santacasasp.org.br; ^2^ Faculty of Medical Sciences of Santa Casa de São Paulo, São Paulo, Brazil; ^3^ Jundiaí Medical School, Jundiaí, Brazil; ^4^ São Caetano do Sul Municipal University, São Caetano do Sul, Brazil

**Keywords:** breast cancer, oxidative stress, selenium, systematic review, zinc

## Abstract

**Background:**

Selenium (Se) and zinc (Zn) are essential micronutrients that play roles in antioxidant defense and the regulation of cell proliferation. Increasing evidence suggests that disturbances in trace element balance may contribute to breast carcinogenesis; however, findings across studies remain inconsistent.

**Objective:**

To evaluate the association between circulating and tissue Se and Zn levels and breast cancer.

**Methods:**

A systematic review and meta‐analysis were conducted according to PRISMA 2020 and MOOSE recommendations. Searches were performed in MedLine, EMBASE, and LILACS from database inception until April 15, 2026. Case–control studies comparing selenium and zinc levels between women with breast cancer and control groups were eligible. Two independent reviewers performed study selection, data extraction, risk‐of‐bias assessment, and publication bias analysis using Egger’s regression test.

**Results:**

Thirty case–control studies were included. Most studies (83.3%) were classified as high methodological quality according to the Newcastle–Ottawa Scale. Meta‐analyses revealed significantly lower selenium levels in plasma (MD = −12.10 μg/L; 95% CI: −17.54 to −6.65; *p* < 0.0001), selenium in nails (MD = −0.02 μg/g; 95% CI: −0.04 to −0.01; *p* = 0.006), and zinc in plasma (MD = −0.33; 95% CI: −0.47 to −0.19; *p* < 0.00001) in breast cancer patients compared with controls.

**Conclusions:**

The pooled findings indicate an inverse association between breast cancer and selenium levels measured in plasma and nails, as well as plasma zinc concentrations. Nevertheless, interpretation should remain cautious because all included studies had observational designs, with marked heterogeneity and potential residual confounding. Lower circulating selenium and zinc levels, together with reduced selenium concentrations in nails, were associated with breast cancer occurrence. Additional prospective studies are required to clarify causality and determine the clinical significance of these associations.

## 1. Introduction

Breast cancer is the most prevalent malignant neoplasm among women worldwide and represents one of the main global public health challenges. Its development is influenced by a complex interaction of genetic, hormonal, environmental, and nutritional factors [1]. Among the various factors that influence tumor development and progression, the presence and balance of micronutrients in the body have attracted increasing scientific interest and may contribute to carcinogenesis [2].

In this context, biological mechanisms—particularly oxidative stress, resulting from an imbalance between the production of reactive oxygen species (ROS) and the capacity of antioxidant systems—have been implicated in all stages of breast carcinogenesis [3]. Selenium contributes to antioxidant protection through its incorporation into selenoproteins, including glutathione peroxidases, which participate in the neutralization of oxidative damage. Zinc, in turn, acts as a cofactor for over 300 enzymes and is essential for processes such as DNA synthesis, cell division, and regulation of apoptosis [4]. Deficiency of these micronutrients can compromise antioxidant defenses and genomic integrity, theoretically increasing susceptibility to breast cancer [5].

Several observational studies have investigated the association between body levels of Se and Zn—measured in different biological matrices such as plasma, nails, and hair—and breast cancer risk. However, the results are sometimes inconsistent or vary in magnitude [6].

Given the biological relevance of these micronutrients and their potential impact on breast cancer, this review aims to provide an up‐to‐date synthesis of the scientific literature, incorporating recent studies, analyzing multiple biological matrices, and critically evaluating the quality of the evidence and the heterogeneity of the findings. Thus, it seeks to contribute to a more comprehensive understanding of the association between the body status of these minerals and breast cancer.

## 2. Methods

This systematic review and meta‐analysis was developed following the PRISMA 2020 statement [7] and reported according to MOOSE (Meta‐analysis of Observational Studies in Epidemiology) recommendations for meta‐analyses of observational studies [8]. This study is registered on the PROSPERO platform (registration number: CRD420261359670).

### 2.1. Eligibility Criteria

Case–control, cohort, and randomized controlled studies published up to April 15, 2026, were considered eligible. However, only case–control studies met the inclusion criteria. These studies compared selenium (Se) and/or zinc (Zn) levels in serum, nails, and/or hair between women with breast cancer (cases) and women without breast cancer (controls). Other designs, such as cross‐sectional studies, case series, and case reports, were excluded due to their methodological limitations, including lower levels of evidence and the lack of ability to establish temporal relationships or adequately assess the associations relevant to the study objective.

The PECO criteria (Population, Exposure/Intervention, Comparison, and Outcome) were used to search for and identify eligible studies. A detailed table of the search terms used in each database is presented in Table 1.

**TABLE 1:** PECO criteria.

PECO element	Characteristics
Population	Women aged > 18 years diagnosed with primary breast cancer, confirmed by clinical, mammography, fine needle aspiration biopsy (FNAB), histopathological or registry‐based diagnosis. Studies including breast cancer recurrence were excluded.
Exposure	Selenium and/or zinc levels in plasma, hair, and nails, measured using validated laboratory methods. Biomarker measurements were considered when performed at or near diagnosis, prior to treatment, when such information was available.
Comparison	Women aged > 18 years without a diagnosis of breast cancer or with a diagnosis of benign breast disease who attend the hospital where the studies were conducted.
Outcome	Differences in Se and Zn levels between groups and their association with breast cancer presence.

### 2.2. Search Strategy and Databases

A systematic search was conducted in MedLine (via PubMed), EMBASE, and LILACS, supplemented by manual searching. An initial search strategy was developed based on key concepts related to breast cancer, selenium, and zinc. This core strategy combined controlled vocabulary terms (e.g., MeSH and Emtree) and free‐text terms using Boolean operators (AND, OR). The same search strategy was applied across all databases: (Breast Neoplasm OR Breast Neoplasms OR Breast Tumors OR Breast Tumor OR Mammary Neoplasms OR Mammary Neoplasm OR Mammary Carcinoma OR Mammary Carcinomas OR Breast Cancer OR Cancer of Breast OR Mammary Cancer OR Malignant Neoplasm of Breast OR Malignant Tumor of Breast OR Breast Carcinoma OR Cancer of the Breast) AND (Selenium OR Zinc). Differences observed in the final search strings reflect the automatic processing of each database, including variations in indexing systems, controlled vocabularies, and search syntax, rather than intentional modifications. No language restrictions were applied during the literature search.

Although the conceptual structure of the search strategy remained consistent across databases, minor adaptations were required to accommodate differences in indexing systems and search syntax. Complete search strategies for each database are presented in Supporting Information 1.

Prior to data extraction, all eligible studies were checked for retractions, corrections, or editorial expressions of concern using the Retraction Watch Database [9] and journal websites.

### 2.3. Data Extraction

Two independent reviewers selected the studies according to the aforementioned search strategy. Titles, abstracts, and full texts of each article were screened. Discrepancies were resolved by consensus or by a third reviewer when necessary. Extracted data included study identification, design, population, biological matrix analyzed, analytical method, and mean levels and standard deviation of Se/Zn.

### 2.4. Risk of Bias Assessment

The Newcastle–Ottawa Scale (NOS) was used for assessing case–control studies. Study quality was classified as high (7–9 points), moderate (4–6), or low (0–3). This scale evaluates three domains: selection, comparability, and exposure. For the comparability domain, studies were awarded one or two stars based on adjustment for key confounders, including age, body mass index, and lifestyle factors (e.g., smoking or dietary intake), when reported.

Furthermore, publication bias was assessed using Egger’s regression test, which evaluates the skewness of the funnel plot through linear regression between standardized effect estimates and their precision (inverse standard error). A *p* value < 0.05 was considered indicative of potential small study effects or publication bias. The funnel plots were also visually inspected for skewness. Egger’s test was not applied when fewer than ten studies were available for a given outcome [10].

### 2.5. Data Analysis and Statistical Synthesis

The extracted information was systematically compiled into structured tables containing study identification, biological matrix evaluated, micronutrient analyzed, number of participants in case and control groups, and the corresponding mean concentrations and standard deviations of selenium and zinc measurements. Continuous outcomes were synthesized using weighted mean differences (WMDs), since most studies reported selenium and zinc concentrations using comparable measurement scales. When original studies presented results as medians, quartiles, or ranges rather than means and standard deviations, statistical conversion methods proposed by Luo et al. (2018) [11] and Wan et al. (2014) [12] were applied to estimate these parameters. In studies where numerical information was available only in graphical format, values were extracted using the WebPlotDigitizer software.

For quantitative synthesis, studies were categorized according to the micronutrient evaluated (selenium or zinc) and the biological specimen analyzed (plasma, hair, or nails). Studies reporting serum and plasma selenium or zinc concentrations were analyzed together as circulating biomarkers because of their biological comparability. Meta‐analyses were performed using Review Manager Version 5.3. Both fixed‐effect and random‐effects approaches were explored, considering the expected methodological and clinical variability among studies.

Pooled estimates were expressed with corresponding 95% confidence intervals, and statistical heterogeneity was quantified using the *I* ^2^ statistic. Heterogeneity values above 50% were interpreted as substantial, whereas values exceeding 75% were considered indicative of considerable inconsistency among studies.

## 3. Results

### 3.1. Study Selection

The literature search identified 11,453 potentially relevant records published between 1935 and April 15, 2026, including 3898 from PubMed, 7437 from Embase, and 118 from LILACS. Following title and abstract screening, 11,341 records were excluded because they did not meet the predefined eligibility criteria.

An additional four studies available only as conference abstracts were excluded due to insufficient outcome data for extraction and analysis. Subsequently, 108 full‐text articles underwent detailed eligibility assessment. Among these, 78 studies were excluded for the following reasons: one publication corresponded to an erratum, 70 did not report outcomes relevant to the review question, and seven presented incomplete or inconsistent outcome data that precluded inclusion in the quantitative synthesis.

**FIGURE 1:**
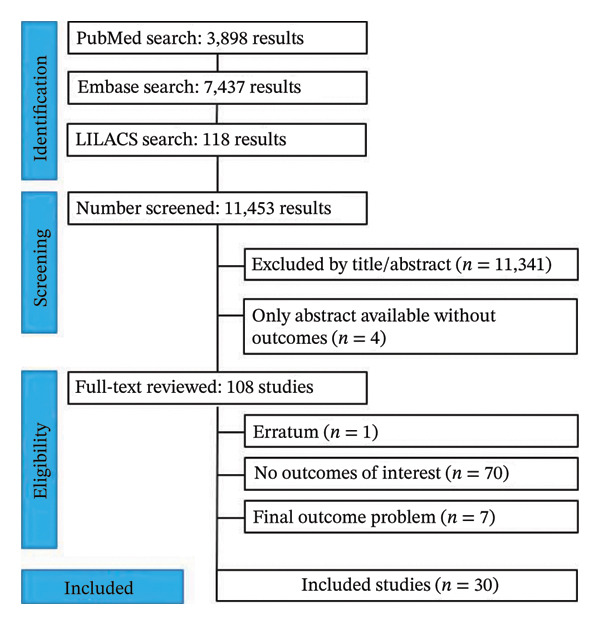
PRISMA flow diagram of study selection.

Ultimately, 30 case–control studies fulfilled the eligibility criteria and were included in the systematic review and meta‐analysis (Figure 1). To ensure research integrity, all included articles were additionally screened through the Retraction Watch Database and journal websites. No retractions, corrections compromising validity, or editorial expressions of concern were identified for the included studies [9].

### 3.2. Study Characteristics

The studies included in this review all employed a case–control design. Selenium and/or zinc concentrations were evaluated in different biological specimens, including serum, hair, and nail samples, with studies reporting mean values and corresponding standard deviations for both breast cancer patients and comparison groups.

The composition of the control groups varied across studies, consisting either of healthy women or individuals diagnosed with benign breast conditions. Information regarding ICD‐based breast cancer classification was not consistently available in the included publications.

Population characteristics also differed between studies. While some investigations focused exclusively on premenopausal women, others included postmenopausal participants or mixed populations, each analyzed against their respective control groups. In addition, several studies reported separate subgroup analyses within the same publication, generating more than one independent comparison for quantitative synthesis. Detailed characteristics of the included studies are summarized in Table 2.

**TABLE 2:** Characteristics of the included studies.

Title	Study	Molecule (Se/Zn)	Sample studied	Participants	Patient group	Control group	Subgroups
Abdollahi A, 2015	Case–control	Zn	Serum	87	38	49	—

Adeoiti ML, 2015	Case–control	Se	Serum	60	30	30	—
		Zn	Serum	60	30	30	—

Ajayi GO, 2011	Case–control	Zn	Serum	18	9	9	—

Anatoly VS, 2020	Case–control	Se	Serum	207	107	100	—
		Zn	Serum	207	107	100	—
		Se	Hair	207	107	100	—
		Zn	Hair	207	107	100	—

Basu TK, 1989	Case–control	Se	Serum	38	19	119	—

Cavallo F, 1990	Case–control	Zn	Serum	418	206	212	Milan
		Zn	Serum	93	47	46	Montpellier

Choi R, 2018	Case–control	Se	Serum	287	150	137	Discovery cohort
		Zn	Serum	142	79	63	Validation cohort

Cihan YB, 2012	Case–control	Se	Hair	104	52	52	—
		Zn	Hair	104	52	52	—

Feng JF, 2011	Case–control	Se	Serum	76	56	20	—
		Zn	Serum	76	56	20	—

Gerber M, 1991	Case–control	Se	Serum	97	47	50	—
		Zn	Serum	98	48	50	—
		Se	Nail	64	25	39	—

Gupta SK, 1991	Case–control	Zn	Serum	65	35	30	—

Hardell L, 1993	Case–control	Se	Serum	413	278	135	—

Hashemi SM, 2017	Case–control	Zn	Serum	300	142	158	—

Huynh PT, 2020	Case–control	Se	Nail	69	34	35	—

Krsnjavi H, 1990	Case–control	Se	Serum	76	33	43	—

Kuo HW, 1999	Case–control	Se	Serum	50	25	25	—
		Zn	Serum	50	25	25	—

Männistö S, 2000	Case–control	Se	Nail	280	112	168	Premenopausal
		Se	Nail	442	177	265	Postmenopausal

Memon AU, 2006	Case–control	Zn	Serum	80	30	50	—
		Zn	Hair	80	30	50	—

Moradi M, 2008	Case–control	Se	Serum	90	45	45	—

Overvad K, Gron P 1991	Case–control	Se	Serum	159	66	93	—

Overvad K, Wang DY 1991	Case–control	Se	Serum	184	46	138	—

Pala V, 2022	Case–control	Zn	Serum	974	487	487	—

Piccinini L, 1996	Case–control	Se	Serum	60	38	22	—
		Zn	Serum	60	38	22	—
		Se	Hair	60	38	22	—
		Zn	Hair	60	38	22	—

Schrauzer GN, 1985	Case–control	Se	Serum	50	25	25	American
		Se	Serum	104	79	25	Japanese

Singh P, 2005	Case–control	Se	Serum	320	160	160	—

Suzana S, 2009	Case–control	Se	Hair	48	12	36	—

Tinoco‐Veras CM, 2011	Case–control	Zn	Serum	55	29	26	—

Ujiie S, 2002	Case–control	Se	Serum	1563	313	1250	—

Van Noord PA, 1993	Case–control	Se	Nail	337	67	270	—

Van’t veer P, 1990	Case–control	Se	Serum	243	92	151	—
		Se	Nail	360	124	236	—

### 3.3. Risk of Bias

According to the NOS assessment, 25 of the 30 included studies (83.3%) were classified as having high methodological quality (NOS score ≥ 7). The most frequent methodological concerns were related to the selection of control groups and the limited adjustment for potential confounding factors.

Differences in analytical and laboratory procedures across studies may also have contributed to the heterogeneity observed in micronutrient measurements (Table 3). In addition, the possibility of publication bias was investigated using Egger’s regression test, and the corresponding findings are summarized in Table 4.

**TABLE 3:** Risk of bias assessment (Newcastle–Ottawa Scale).

Newcastle–Ottawa Scale				
Study	Selection	Comparability	Exposure	Total
Abdollahi A, 2015	3	2	3	8
Adeoiti ML, 2015	2	2	3	7
Ajayi GO, 2011	3	2	3	8
Anatoly VS, 2020	4	2	3	9
Basu TK, 1989	3	2	3	8
Cavallo F, 1990	3	2	3	8
Choi R, 2018	4	1	3	8
Cihan YB, 2012	2	2	3	7
Feng JF, 2011	3	2	3	8
Gerber M, 1991	3	2	3	8
Gupta SK, 1991	3	0	3	6
Hardell L, 1993	1	2	3	6
Hashemi SM, 2017	4	1	3	8
Huynh PT, 2020	3	1	3	7
Krsnjavi H, 1990	3	2	3	8
Kuo HW, 1999	4	2	3	9
Männistö S, 2000	1	2	3	6
Memon AU, 2006	2	2	3	7
Moradi M, 2008	2	2	3	7
Overvad K, Gron P 1991	4	1	3	8
Overvad K, Wang DY 1991	4	1	1	6
Pala V, 2022	4	2	3	9
Piccinini L, 1996	2	2	3	7
Schrauzer GN, 1985	1	1	3	5
Singh P, 2005	3	2	3	8
Suzana S, 2009	3	2	3	8
Tinoco‐Veras CM, 2011	3	2	3	8
Ujiie S, 2002	3	2	3	8
Van Noord PA, 1993	3	2	3	8
Van’t veer P, 1990	3	2	3	8

**TABLE 4:** Egger’s regression test.

Egger’s regression test	
Plasma Se *N* = 19	Test result: *t* = −2.92, df = 17, *p* = 0.0095; bias estimate: −3.5057 (SE = 1.1993)
Plasma Zn *N* = 14	Test result: *t* = −2.48, df = 12, *p* = 0.0287; bias estimate: −12.7831 (SE = 5.1451)
Nail Se *N* = 7	Number of studies (*k* = 7) was below the minimum required to assess small‐study effects (k.min = 10).
Hair Se *N* = 4	Number of studies (*k* = 4) was below the minimum required to assess small‐study effects (k.min = 10).
Hair Zn *N* = 4	Number of studies (*k* = 4) was below the minimum required to assess small‐study effects (k.min = 10).

**FIGURE 2:**
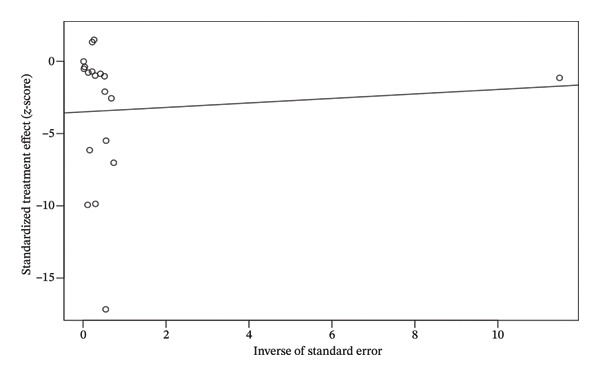
Funnel plot of studies evaluating plasma selenium levels: inverse standard error versus standardized treatment effect (*z*‐score).

**FIGURE 3:**
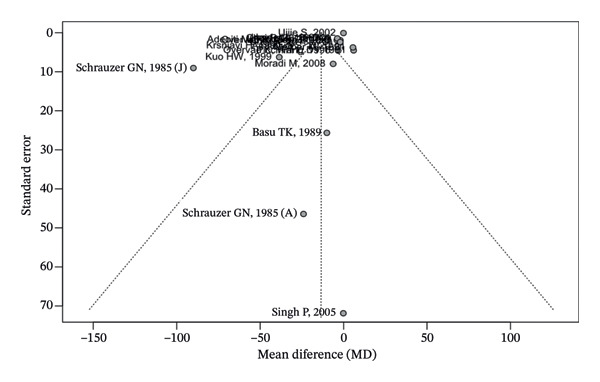
Funnel plot of studies evaluating plasma selenium levels: standard error versus mean difference.

**FIGURE 4:**
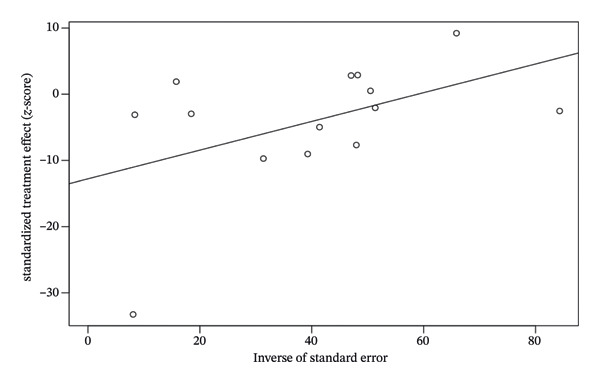
Funnel plot of studies evaluating plasma zinc levels: inverse standard error versus standardized treatment effect (*z*‐score).

**FIGURE 5:**
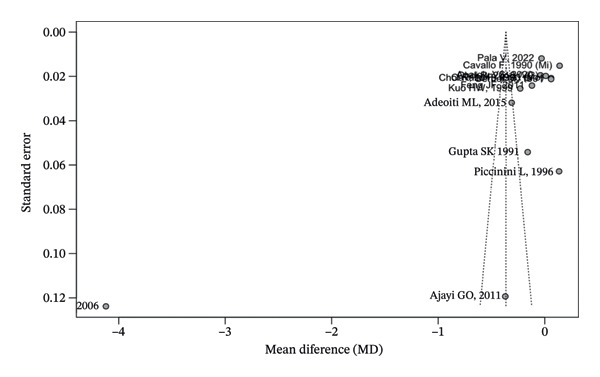
Funnel plot of studies evaluating plasma zinc levels: standard error versus mean difference.

The presence of publication bias was investigated through visual inspection of funnel plots and by applying Egger’s regression test in analyses including a sufficient number of studies (Figures 2–5). In analyses with enough studies, statistically significant evidence of funnel plot asymmetry was observed.

Specifically, in the analysis of plasma Se, Egger’s test showed a significant result (*t* = −2.92; df = 17; *p* = 0.0095), with a bias estimate of −3.51 (standard error = 1.20). A similar pattern was identified in the plasma zinc analysis (*t* = −2.48; df = 12; *p* = 0.0287), with a bias estimate of −12.78 (standard error = 5.15) (Table 4). These findings may indicate the presence of small‐study effects, in which effect estimates appear to vary according to study size.

On the other hand, for subgroups in which the number of studies was below the recommended threshold (*n* < 10), including analyses of hair samples (*n* = 4 for zinc and selenium) and nail samples (*n* = 7), Egger’s test was not considered applicable due to low statistical power. Consequently, the assessment of funnel plot asymmetry and potential publication bias for these subgroups was considered unreliable.

In analyses performed with plasma data, where the number of studies was adequate (zinc: *n* = 14; selenium: *n* = 19), the results indicated evidence of asymmetry, reinforcing the possibility of publication bias or other effects related to sample size. Studies with high variability in plasma zinc levels were excluded in the Egger test.

### 3.4. Synthesis of Results

#### 3.4.1. Plasma Selenium

**FIGURE 6:**
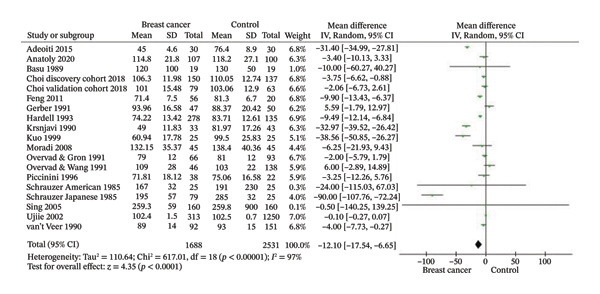
Forest plot: plasma selenium.

The meta‐analysis evaluating plasma selenium concentrations included 17 studies comprising 19 independent subgroups, with a total of 1688 women with breast cancer and 2531 controls (4219 participants overall). Pooled analysis demonstrated that individuals with breast cancer presented significantly lower plasma selenium levels compared with controls (MD = −12.10 μg/L; 95% CI: −17.54 to −6.65; *p* < 0.0001). Considerable heterogeneity was identified across studies (*I* ^2^ = 97%) (Figure 6).

#### 3.4.2. Hair Selenium

**FIGURE 7:**
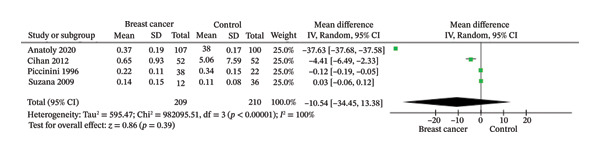
Forest plot: hair selenium.

The analysis of selenium concentrations measured in hair samples included four studies comprising 209 breast cancer cases and 210 control participants, totaling 419 individuals. The pooled results did not demonstrate a statistically significant difference between groups (MD = −10.54 μg/g; 95% CI: −34.45 to 13.38; *p* = 0.39). A very high degree of heterogeneity was observed among the included studies (*I* ^2^ = 100%) (Figure 7).

#### 3.4.3. Selenium in Nails

**FIGURE 8:**
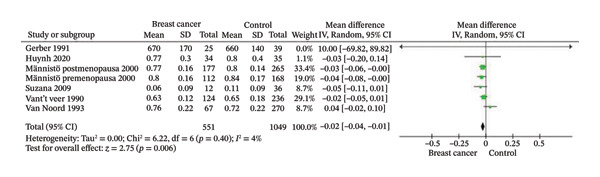
Forest plot: selenium in nails.

The meta‐analysis of nail selenium concentrations incorporated six studies comprising seven independent subgroups, with a total of 551 women with breast cancer and 1049 controls (1600 participants overall). Compared with the control groups, patients with breast cancer exhibited significantly lower selenium concentrations in nail samples (MD = −0.02 μg/g; 95% CI: −0.04 to −0.01; *p* = 0.006). Low statistical heterogeneity was observed across the included studies (*I* ^2^ = 4%) (Figure 8).

#### 3.4.4. Zinc in Plasma

**FIGURE 9:**
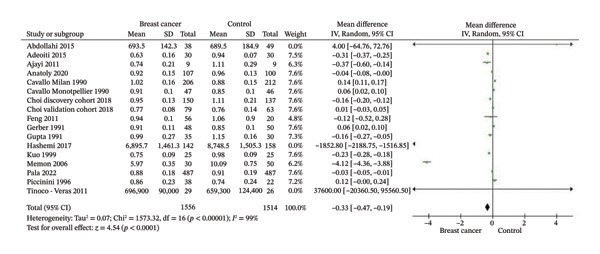
Forest plot: zinc in plasma.

The pooled analysis of plasma zinc concentrations included 15 studies comprising 17 independent subgroups, corresponding to 1556 breast cancer cases and 1514 controls, for a total of 3070 participants. Women with breast cancer showed significantly reduced plasma zinc levels compared with control groups (MD = −0.33; 95% CI: −0.47 to −0.19; *p* < 0.00001). Considerable heterogeneity was detected among the included studies (*I* ^2^ = 99%) (Figure 9).

#### 3.4.5. Zinc in Hair

**FIGURE 10:**
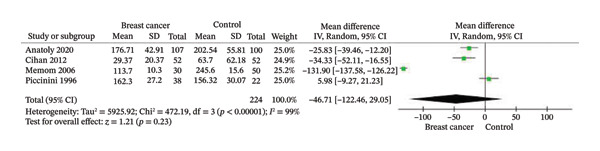
Forest plot: zinc in hair.

Four studies evaluating zinc concentrations in hair samples were included in the quantitative synthesis, comprising 227 women with breast cancer and 224 controls (451 participants in total). The pooled analysis did not identify a statistically significant difference in hair zinc levels between cases and controls (MD = −46.71 μg/g; 95% CI: −122.46 to 29.05; *p* = 0.23). A high degree of between‐study heterogeneity was observed (*I* ^2^ = 99%) (Figure 10).

## 4. Discussion

This updated systematic review and meta‐analysis, including 30 case–control studies, supports the presence of an inverse association between circulating selenium and zinc concentrations and breast cancer. Lower selenium levels measured in nails, which may reflect medium‐ and long‐term exposure, were also associated with the disease. These results are consistent with previous meta‐analyses [6, 13, 14] and with the recognized biological functions of these micronutrients in breast carcinogenesis [4–6].

The associations for plasma selenium, plasma zinc, and nail selenium remained consistent after sensitivity analyses excluding outlier or lower‐quality studies. Nevertheless, despite the statistical significance of the pooled estimates, the evidence should be interpreted cautiously because all included studies had observational designs and considerable variability was identified across analyses.

The available evidence is derived predominantly from case–control studies, limiting the ability to establish temporal relationships and causal interpretation. Reverse causality remains possible, since breast cancer itself, related metabolic alterations, or treatment effects may influence selenium and zinc concentrations [15]. Consequently, the observed associations should be interpreted cautiously and not as definitive evidence of causation.

Important between‐study variability (*I* ^2^ > 68% in most analyses) also represents a relevant limitation. Multiple factors may have contributed to this variability, including differences in laboratory methods, dietary and genetic diversity among populations, selection of controls, and adjustment for confounding variables. Geographic variation in zinc exposure, particularly dietary intake and lifestyle, as well as genetic polymorphisms affecting micronutrient metabolism, may partially explain the observed differences between populations [14]. The very high heterogeneity identified in hair analyses (*I* ^2^ > 97%) suggests that this biological matrix may provide less consistent results in this context. Although subgroup analyses or meta‐regression could help explore these sources of heterogeneity, such analyses were not feasible because of inconsistent reporting across the primary studies.

Although lower plasma and nail selenium concentrations were associated with breast cancer, the clinical relevance of these differences remains uncertain. Moderate reductions in selenium availability may impair antioxidant enzyme activity and contribute to oxidative stress and genomic instability; however, clinically meaningful thresholds remain unclear. Future studies should correlate biomarker levels with oxidative stress markers, inflammation, and prognosis.

The possibility of publication bias should also be acknowledged. Although gray literature was considered, studies with null findings may remain unpublished. Funnel plot inspection and Egger’s regression analysis suggested some degree of asymmetry in plasma‐based analyses; however, interpretation of these findings should be cautious because asymmetry tests may have limited reliability in analyses with high inconsistency across studies or a reduced number of studies [10].

Overall, these methodological considerations indicate that the observed associations are biologically plausible, although current evidence remains insufficient to establish a definitive causal relationship between selenium, zinc, and breast cancer.

### 4.1. Context and Comparison

The present findings regarding plasma selenium and zinc concentrations are generally in agreement with previously published meta‐analyses [6, 13, 14]. However, an important distinction of the current review is the separate evaluation of different biological matrices, allowing identification of distinct association patterns across plasma, nails, and hair samples. In the present analysis, significant associations were consistently observed for plasma and nail biomarkers, whereas hair measurements did not demonstrate comparable findings. This review expands previous evidence through matrix‐specific analyses and sensitivity assessments. In addition, a meta‐analysis evaluating dietary selenium and zinc intake in Chinese populations [6] similarly reported inverse associations with breast cancer risk, suggesting that both dietary exposure and biological micronutrient status may be relevant to disease development, although biomarker concentrations do not always directly reflect dietary intake.

### 4.2. Geographic Variation and Nutritional Context

Selenium exposure differs substantially across geographic regions, largely because soil composition directly influences food selenium content and dietary intake. This regional variability may partially explain the heterogeneity identified among studies, particularly because populations living in areas with lower selenium availability could present stronger associations between selenium deficiency and breast cancer risk. Furthermore, differences in dietary habits, nutritional patterns, and cultural food practices may also contribute to variations in micronutrient status, emphasizing the importance of considering geographic and nutritional context when interpreting these findings.

### 4.3. Limitations and Strengths

Important limitations include methodological variability between studies, differences in control selection, and incomplete adjustment for confounding factors. Histological subtype and histopathological classification could not be consistently evaluated because these variables were not uniformly reported. Major strengths include adherence to PRISMA and MOOSE recommendations, comprehensive literature searches, formal risk‐of‐bias assessment, sensitivity analyses, and the inclusion of multiple biological matrices.

### 4.4. Implications and Future Directions

Current evidence, while suggestive, is insufficient to recommend Se or Zn supplementation for primary breast cancer prevention. Research priorities should focus on conducting large‐scale prospective cohort studies with standardized collection of biological samples before diagnosis, measurement of multiple Se/Zn status biomarkers, detailed dietary assessment, and rigorous adjustment for confounders. Such studies are essential to better clarify temporal relationships. Well‐designed randomized clinical trials are needed to evaluate the impact of Se/Zn interventions but should be based on solid hypotheses. Meanwhile, maintaining adequate micronutrient status through a varied and balanced diet remains a fundamental public health recommendation. Future studies should also explore sources of heterogeneity through meta‐regression or predefined subgroup analyses, particularly considering geographic variation, menopausal status, analytical techniques, and control selection.

Although previous meta‐analyses have explored the association between selenium, zinc, and breast cancer, this study provides several methodological advances. First, we performed separate analyses according to biological matrices (plasma, nails, and hair), allowing a more precise interpretation of exposure windows. Second, we applied sensitivity analyses excluding outliers and lower‐quality studies, improving robustness. Third, we included more recent studies, expanding the sample size and strengthening statistical power. These methodological refinements contribute to a more nuanced understanding of the relationship between micronutrient status and breast cancer.

In summary, this review contributes updated evidence, matrix‐specific analyses, and additional sensitivity assessments, providing a broader and more critical evaluation of the association between selenium, zinc, and breast cancer risk. Nevertheless, the inclusion of higher‐level evidence, particularly prospective cohort studies and randomized clinical trials, remains essential for improving causal inference and clarifying the temporal relationship between micronutrient status and breast cancer development.

## 5. Conclusion

This systematic review and meta‐analysis identified inverse associations between breast cancer and reduced concentrations of plasma selenium, plasma zinc, and nail selenium biomarkers. Nevertheless, the current evidence is based mainly on case–control studies and is affected by marked heterogeneity, as well as methodological limitations within the primary studies, which restricts causal interpretation and limits conclusions regarding clinical relevance. Future well‐designed prospective studies are necessary to better clarify the possible role of these micronutrients in breast cancer development.

## Author Contributions

Adrienne Pratti Lucarelli, as the corresponding author and manuscript guarantor, had full access to all of the data in this study and takes complete responsibility for the integrity of the data and the accuracy of the data analysis. Adrienne Pratti Lucarelli: conceptualization; methodology; formal analysis; investigation; data curation; writing–original draft; writing–review and editing; supervision; and project administration. Maria Marta Martins: methodology; validation; writing–review and editing; and supervision. Mariana Mie Teruya: investigation; data curation; and writing–review and editing. Thaís Tiemi Yauti: investigation; data curation; methodology; and writing–review and editing. Daniela Rie Hayashi: data curation; formal analysis; visualization; and writing–review and editing.

## Funding

This study did not receive any specific funding. Open access publishing support was provided by Faculdade de Ciências Médicas da Santa Casa de São Paulo.

## Disclosure

All authors have read and approved the final version of the manuscript.

## Ethics Statement

Ethical approval was not required for this study because it is a systematic review and meta‐analysis based exclusively on data extracted from previously published studies, without access to identifiable individual participant data. No new data were collected, no human participants were directly involved, and no identifiable individual participant information was accessed or analyzed. Therefore, informed consent was also not required.

## Conflicts of Interest

The authors declare no conflicts of interest.

## Supporting Information

Additional supporting information can be found online in the Supporting Information section.

## Supporting information


**Supporting Information 1** Full electronic search strategies. This file provides the complete electronic search strategies for MedLine (via PubMed), EMBASE, and LILACS, including the database‐specific search syntax used in the systematic review.

**Supporting Information 2** MOOSE checklist. Completed Meta‐analysis Of Observational Studies in Epidemiology (MOOSE) checklist reporting the methodological and reporting items addressed in this study.

## Data Availability

The authors confirm that the data supporting the findings of this study are available within the article and/or its supporting information.
