# Supplementary material for: Systematic Review and Meta‐Analysis: Association Between Circulating and Tissue Levels of Selenium and Zinc and Breast Cancer Risk
Source: Breast J. 2026 Jul 2;2026:7448703. doi: 10.1155/tbj/7448703 (PMC13325408; doi:10.1155/tbj/7448703)
Supplement: Supplementary file 1 — Supporting Information 1 Full electronic search strategies. This file provides the complete electronic search strategies for MedLine (via PubMed), EMBASE, and LILACS, including the database‐specific search syntax used in the systematic review. [file TBJ-2026-7448703-s001.docx]

# Supplementary Material 1 – Full Electronic Search Strategies

## Systematic Review and Meta-Analysis: Association Between Circulating and Tissue Levels of Selenium and Zinc and Breast Cancer Risk

Running head: Association Between Levels of Selenium and Zinc and Breast Cancer Risk

Date of last search: April 15, 2026

Databases searched: MedLine (via PubMed), EMBASE, LILACS No database filters were applied during the search.

The same core search strategy was applied across all databases. Differences in the final search strings reflect the automatic processing and indexing systems of each database (e.g., MeSH in PubMed, Emtree in EMBASE, and DeCS in LILACS), rather than intentional modifications by the authors.

MedLine (via PubMed)

(Breast Neoplasm OR Breast Neoplasms OR Breast Tumors OR Breast Tumor OR Mammary Neoplasms OR Mammary Neoplasm OR Mammary Carcinoma OR Mammary Carcinomas OR Breast Cancer OR Cancer of Breast OR Mammary Cancer OR Malignant Neoplasm of Breast OR Malignant Tumor of Breast OR Breast Carcinoma OR Cancer of the Breast) AND (Selenium OR Zinc).

EMBASE

('breast neoplasm'/exp OR 'breast neoplasm' OR (('breast'/exp OR breast) AND ('neoplasm'/exp OR neoplasm)) OR 'breast neoplasms'/exp OR 'breast neoplasms' OR (('breast'/exp OR breast) AND ('neoplasms'/exp OR neoplasms)) OR 'breast tumors'/exp OR 'breast tumors' OR (('breast'/exp OR breast) AND ('tumors'/exp OR tumors)) OR 'breast tumor'/exp OR 'breast tumor' OR (('breast'/exp OR breast) AND ('tumor'/exp OR tumor)) OR 'mammary neoplasms'/exp OR 'mammary neoplasms' OR (mammary AND ('neoplasms'/exp OR neoplasms)) OR 'mammary neoplasm'/exp OR 'mammary neoplasm' OR (mammary AND ('neoplasm'/exp OR neoplasm)) OR 'mammary carcinoma'/exp OR 'mammary carcinoma' OR (mammary AND ('carcinoma'/exp OR carcinoma)) OR 'mammary carcinomas' OR (mammary AND carcinomas) OR 'breast cancer'/exp OR 'breast cancer' OR (('breast'/exp OR breast) AND ('cancer'/exp OR cancer)) OR 'cancer of breast' OR (('cancer'/exp OR

cancer) AND of AND ('breast'/exp OR breast)) OR 'mammary cancer'/exp OR 'mammary cancer' OR (mammary AND ('cancer'/exp OR cancer)) OR 'malignant neoplasm of breast' OR (malignant AND ('neoplasm'/exp OR neoplasm) AND of AND ('breast'/exp OR breast)) OR 'malignant tumor of breast' OR (malignant AND ('tumor'/exp OR tumor) AND of AND ('breast'/exp OR breast)) OR 'breast carcinoma'/exp OR 'breast carcinoma' OR (('breast'/exp OR breast) AND ('carcinoma'/exp OR carcinoma)) OR 'cancer of the breast'/exp OR 'cancer of the breast' OR (('cancer'/exp OR cancer) AND of AND the AND ('breast'/exp OR breast))) AND ('selenium'/exp OR selenium OR 'zinc'/exp OR zinc)

LILACS (via BVS)

tw:("Breast Neoplasm" OR "Breast Neoplasms" OR "Breast Tumors" OR "Breast Tumor" OR "Mammary Neoplasms" OR "Mammary Neoplasm" OR "Mammary Carcinoma" OR "Mammary Carcinomas" OR "Breast Cancer" OR "Cancer of Breast" OR "Mammary Cancer" OR "Malignant Neoplasm of Breast" OR "Malignant Tumor of Breast" OR "Breast Carcinoma" OR "Cancer of the Breast") AND tw:("Selenium" OR "Zinc")

Additional Search Methods

Manual screening of reference lists from all included studies

Screening of relevant systematic reviews to identify additional eligible studies, when applicable

No date restrictions were applied

Study Selection Notes

No study design filters were applied during database searches to maximize sensitivity. Study design eligibility (case-control studies only) was determined during the screening phase according to predefined inclusion and exclusion criteria.
