# Supplementary material for: Systematic Review and Meta‐Analysis: Association Between Circulating and Tissue Levels of Selenium and Zinc and Breast Cancer Risk
Source: Breast J. 2026 Jul 2;2026:7448703. doi: 10.1155/tbj/7448703 (PMC13325408; doi:10.1155/tbj/7448703)
Supplement: Supplementary file 2 — Supporting Information 2 MOOSE checklist. Completed Meta‐analysis Of Observational Studies in Epidemiology (MOOSE) checklist reporting the methodological and reporting items addressed in this study. [file TBJ-2026-7448703-s002.docx]

# MOOSE Checklist for Meta-analyses of Observational Studies

| **Item No** | **Recommendation** | **Reported on Page No** |
| --- | --- | --- |
| Reporting of background should include | | |
| 1 | Problem definition | 4 |
| 2 | Hypothesis statement | 4 |
| 3 | Description of study outcome(s) | 16 - 18 |
| 4 | Type of exposure or intervention used | 5 - 6, Table 1 |
| 5 | Type of study designs used | 5 |
| 6 | Study population | 5 - 6 |
| Reporting of search strategy should include | | |
| 7 | Qualifications of searchers (eg, librarians and investigators) | 7 |
| 8 | Search strategy, including time period included in the synthesis and key words | 6, Supplementary  Material 1 |
| 9 | Effort to include all available studies, including contact with authors | 6, Supplementary  Material 1 |
| 10 | Databases and registries searched | 6, Supplementary  Material 1 |
| 11 | Search software used, name and version, including special features used (eg, explosion) | 6, Supplementary  Material 1 |
| 12 | Use of hand searching (eg, reference lists of obtained articles) | 6, Supplementary  Material 1 |
| 13 | List of citations located and those excluded, including justification | 8, Figure 1 |
| 14 | Method of addressing articles published in languages other than English | 6 |
| 15 | Method of handling abstracts and unpublished studies | 8 |
| 16 | Description of any contact with authors | - |
| Reporting of methods should include | | |
| 17 | Description of relevance or appropriateness of studies assembled for assessing the  hypothesis to be tested | 9 - 12, Table 2 |
| 18 | Rationale for the selection and coding of data (eg, sound clinical principles or  convenience) | 12 - 16, Tables 3 - 4 |
| 19 | Documentation of how data were classified and coded (eg, multiple raters, blinding and  interrater reliability) | 7 - 8 |
| 20 | Assessment of confounding (eg, comparability of cases and controls in studies where appropriate) | 7, 12 - 13, Table 3 |
| 21 | Assessment of study quality, including blinding of quality assessors, stratification or regression on possible predictors of study results | 7, 12 - 16, Tables 3 -  4, Figures 2 - 5 |
| 22 | Assessment of heterogeneity | 8, 16 - 19 |
| 23 | Description of statistical methods (eg, complete description of fixed or random effects models, justification of whether the chosen models account for predictors of study results, dose-response models, or cumulative meta-analysis) in sufficient detail to be  replicated | 7 - 8 |
| 24 | Provision of appropriate tables and graphics | Tables 1 - 4, Figures 1  - 10 |
| Reporting of results should include | | |
| 25 | Graphic summarizing individual study estimates and overall estimate | 16 - 19 |

| 26 | Table giving descriptive information for each study included | Table 2 |
| --- | --- | --- |

| 27 | Results of sensitivity testing (eg, subgroup analysis) | 19 - 20 |
| --- | --- | --- |
| 28 | Indication of statistical uncertainty of findings | 16 - 18 |

| **Item No** | **Recommendation** | **Reported on Page No** |
| --- | --- | --- |
| Reporting of discussion should include | | |
| 29 | Quantitative assessment of bias (eg, publication bias) | 12 - 16, Tables 3 - 4,  Figures 2 - 5 |
| 30 | Justification for exclusion (eg, exclusion of non-English language citations) | 5 - 6, 8 |
| 31 | Assessment of quality of included studies | 7, 12 - 13, Table 3 |
| Reporting of conclusions should include | | |
| 32 | Consideration of alternative explanations for observed results | 19 - 20 |
| 33 | Generalization of the conclusions (ie, appropriate for the data presented and within the  domain of the literature review) | 19 - 21 |
| 34 | Guidelines for future research | 20 - 21 |
| 35 | Disclosure of funding source | 22 |

*From*: Stroup DF, Berlin JA, Morton SC, et al, for the Meta-analysis Of Observational Studies in Epidemiology (MOOSE) Group. Meta-analysis of Observational Studies in Epidemiology. A Proposal for Reporting. *JAMA*.

2000;283(15):2008-2012. doi: 10.1001/jama.283.15.2008.
